# The 3D Genome Browser: a web-based browser for visualizing 3D genome organization and long-range chromatin interactions

**DOI:** 10.1186/s13059-018-1519-9

**Published:** 2018-10-04

**Authors:** Yanli Wang, Fan Song, Bo Zhang, Lijun Zhang, Jie Xu, Da Kuang, Daofeng Li, Mayank N. K. Choudhary, Yun Li, Ming Hu, Ross Hardison, Ting Wang, Feng Yue

**Affiliations:** 1 Bioinformatics and Genomics Program, The Pennsylvania State University, University Park, State College, PA 16802 USA; 2 Department of Biochemistry and Molecular Biology, College of Medicine, The Pennsylvania State Hershey, Hershey, PA 17033 USA; 3 Department of Computer and Information Science, University of Pennsylvania, Philadelphia, PA 19104 USA; 4 Department of Genetics, The Edison Family Center for Genome Sciences and Systems Biology, Washington University School of Medicine, St. Louis, MO 63108 USA; 5 Department of Genetics, University of North Carolina, Chapel Hill, NC 27599 USA; 6 Department of Biostatistics, University of North Carolina, Chapel Hill, NC 27599 USA; 7 Department of Computer Science, University of North Carolina, Chapel Hill, NC 27599 USA; 8 Department of Quantitative Health Sciences, Lerner Research Institute, Cleveland Clinic Foundation, Cleveland, OH 44195 USA; 9 Center for Computational Biology and Bioinformatics, Huck Institutes of the Life Sciences, The Pennsylvania State University, University Park, State College, PA 16802 USA

## Abstract

Here, we introduce the 3D Genome Browser, http://3dgenome.org, which allows users to conveniently explore both their own and over 300 publicly available chromatin interaction data of different types. We design a new binary data format for Hi-C data that reduces the file size by at least a magnitude and allows users to visualize chromatin interactions over millions of base pairs within seconds. Our browser provides multiple methods linking distal *cis*-regulatory elements with their potential target genes. Users can seamlessly integrate thousands of other omics data to gain a comprehensive view of both regulatory landscape and 3D genome structure.

**Electronic supplementary material:**

The online version of this article (10.1186/s13059-018-1519-9) contains supplementary material, which is available to authorized users.

## Background

The three-dimensional (3D) organization of mammalian genomes plays an essential role in gene regulation [1–4]. At the DNA level, distal regulatory elements such as enhancers have been shown to be in spatial proximity to their target genes. At a larger scale, topologically associating domains (TADs) have been suggested to be the basic unit of mammalian genome organization [5, 6]. Several recent high-throughput technologies based on chromatin conformation capture (3C) [7] have emerged (such as Hi-C [8], ChIA-PET [9], Capture-C [10], Capture Hi-C [11], PLAC-Seq [12], and HiChIP [13]) and have provided an unprecedented opportunity to study the genome spatial organization in a genome-wide fashion.

As the volume of chromatin interaction data keeps increasing, efficient visualization and navigation of these data become a major bottleneck for their biological interpretation. Due to the size and complexity of these interactome data, it is challenging for an individual lab to store and explore them efficiently. To tackle this challenge, several visualization tools have been developed, and each of them has its unique features and limitations. The Hi-C Data Browser [8] was the first web-based query tool that visualizes Hi-C data as heatmaps. Currently, it does not support zoom functionalities and only hosts limited number of datasets. The WashU Epigenome Browser [14, 15] can display both Hi-C and ChIA-PET data, and it also provides access to thousands of epigenomic datasets from the ENCODE and Roadmap Epigenome projects. Due to the large file size of Hi-C matrices, which could reach hundreds of gigabytes, its speed for uploading and exploring Hi-C data is still not optimal. Furthermore, it does not offer an option to display inter-chromosomal interaction data as heatmaps. Users can also explore Hi-C data in Juicebox [16] and Hi-Glass [17] with great speed, but currently, neither of them provide other types of chromatin interaction data, such as Capture Hi-C or ChIA-PET. Delta browser [18] is another visualization tool with many features and can display both physical view of 3D genome modeling and Hi-C data. However, all the aforementioned tools except for the WashU Epigenome Browser only display Hi-C as a heatmap, which is convenient for visualizing large domain structures such as TADs, but may not be the most informative way for visualizing enhancer-promoter interactions.

Here, we present the 3D Genome Browser (www.3dgenome.org), which is a fast web-based browser that allows users to smoothly explore both published and their own chromatin interaction data. Our 3D Genome Browser features six distinct modes that allow users to explore interactome data tailored toward their own needs, from exploring organization of higher-order chromatin structures at domain level to investigating high-resolution enhancer-promoter interactions. Our browser provides convenient zoom and traverse functions in real time and supports queries by gene name, genomic loci, or SNP rsid. In addition, users can easily incorporate their UCSC Genome Browser and the WashU Epigenome Browser sessions and therefore can simultaneously query and supplement chromatin interaction data with thousands of genetic, epigenetic, and phenotypic datasets, including ChIP-Seq and RNA-Seq data from the ENCODE and Roadmap Epigenomics projects. So far, it has been visited by more than 60,000 unique users from 120 countries surpassing over 600,000 page views. In summary, the 3D Genome Browser represents an invaluable resource and ecosystem for the study of chromosomal organization and gene regulation in mammalian genomes.

## Results and Discussion

### Overall design and implementation of the system

**Fig. 1:**
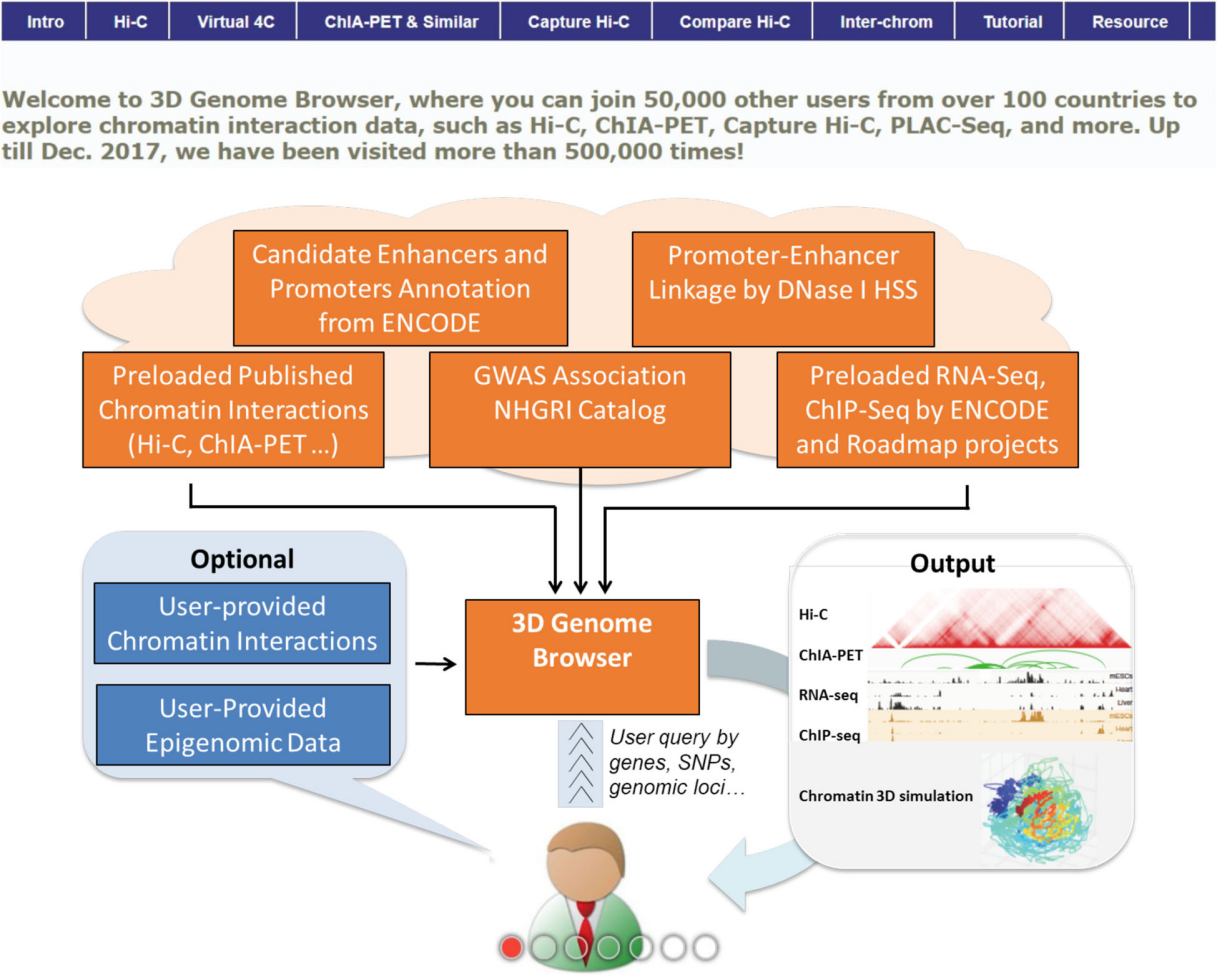
The overall design of the 3D Genome Browser

The overall structure of the 3D Genome Browser is summarized in Fig. 1. Currently, our browser hosts more than 300 chromatin interaction datasets of a variety of different types (Table 1), including Hi-C, ChIA-PET, Capture Hi-C, PLAC-Seq, HiChIP, GAM [19], and SPRITE [20], in both human and mouse across multiple genome assemblies, making it one of the most comprehensive and up-to-date high-quality chromatin interaction data collection (details in Table S1, S2, S3). To increase their impacts and usability, we systematically re-mapped and generated interaction matrices for over 100 Hi-C datasets to the most current genome assembly (GRCh38 and mm10), using the same in-house data processing pipeline.

**Table 1:** Summary of number of datasets available on the 3D Genome Browser

Data type	Samples and conditions	Total datasets
Hi-C	70	288
Virtual 4C, derived from Hi-C	Same as above	Same as above
ChIA-pet	14	14
Capture Hi-C	19	19
HiChIP	2	2
PLAC-Seq	3	3
GAM	1	1
DNase Hi-C	2	2
SPRITE	2	2
Total number	113	331

**Fig. 2:**
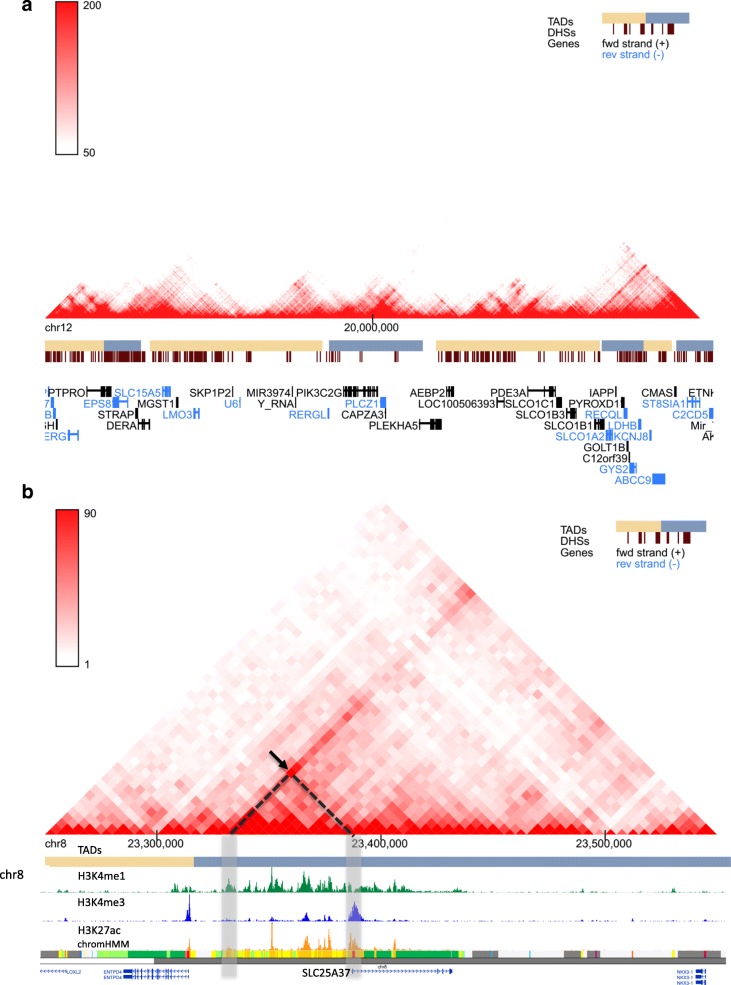
Examples of using the 3D Genome Browser to explore Hi-C data. **a** A 10-Mb region of GM12878 Hi-C interaction map on chr12 (~ 15–25 Mb) at 25-kb resolution. The alternating yellow and blue bars are predicted TADs. The dark red bars are DHS in the same cell type. **b** Hi-C interaction map in K562 cells at 5-kb resolution. The black arrow points to a potential tissue-specific interaction between the *SLC25A37* promoter and a candidate enhancer region (marked by H3K4me1). The ChIP-Seq tracks for histone modifications, and chromHMM are visualized using the WashU Epigenome Browser

One of the important discoveries based on Hi-C data analysis is that the mammalian genomes are organized in mega-base pair chromatin domains, termed topologically associating domains (TADs). Therefore, we adopted the same pipeline from Dixon et al. [5] and systematically predicted TADs in all cell/tissue types (Fig. 2a, orange/blue bars) in our browser. Hi-C data has been shown to contain systematic noises [21]; therefore, we performed ICE (iterative correction and eigenvector decomposition) normalization to all the Hi-C datasets in our browser as well. To further assist users to explore 3D genome organization and gene regulation events simultaneously, we also collected the open chromatin data from the same cell type and display them in the same window (Fig. 2a, red bars). Finally, when users query the chromatin interaction information for a gene, we can also display the expression profiles of this gene across 109 cell/tissue types (Additional file 1: Figure S1), which was uniformly processed by the ENCODE consortium. In summary, for a given genomic loci, our browser can display TADs, chromatin interaction, RNA-Seq, and open chromatin region simultaneously and therefore give our users a comprehensive view of these regions.

To facilitate a user’s unique interest, our 3D Genome Browser features six distinct modes that allow users to explore interactome data, including (1) intra-chromosomal Hi-C contact matrices as heatmaps, coupled with TADs and available genome annotation in the same cell type; (2) inter-chromosomal Hi-C heatmaps: this mode is particularly helpful for visualizing inter-chromosomal interactions and translocations; (3) compare Hi-C matrices: stacked Hi-C heatmaps from different tissues or even different species; (4) virtual 4C: Hi-C data is plotted as an arc for a queried gene or loci (bait), where the center is the bait region. This mode is particularly helpful for revealing chromatin interactions between two individual loci; (5) ChIA-PET or other ChIP-based chromatin interaction data such as PLAC-Seq and HiChIP; (6) Capture Hi-C or other capture-based chromatin interaction data. Below, we will use several examples to demonstrate these options and also illustrate how the 3D Genome Browser can be used to make novel biological discoveries.

### Exploring chromatin interactions using Hi-C data

First, we demonstrate an example of exploring Hi-C data with the 3D Genome Browser for a large genomic region in Fig. 2a. It only takes ~ 5 s to show a 10-Mb region of GM12878 Hi-C interaction map on chr12 (~ 15–25 Mb) at a 25-kb resolution. The alternating yellow and blue bars are predicted TADs using the same in-house pipeline as in Dixon et al. [5]. The dark red vertical bars are DNase I hypersensitive sites (DHS) in the same cell type. Users can also adjust the color scale to reduce the background signals and make the TAD structure more visible.

Identifying cell/tissue-specific chromatin interactions is important, as it has been shown that chromatin structure plays an important role in determining cellular identity [22, 23]. In Fig. 2b, we notice a chromatin interaction in the 5-kb resolution Hi-C contact map in K562 cell line [24] (marked by the black arrow). To interpret biological meaning of this chromatin interaction, we integrated the WashU Epigenome Browser with gene annotation; histone modification H3K4me1, H3K4me3, and H3K27ac; and chromHMM [25] in K562 cells. We found that the two interacting loci are the promoter of *SLC25A37* and a putative enhancer predicted by histone modification patterns and chromHMM (Fig. 2b, vertical gray bar). This putative enhancer has been confirmed to exhibit enhancer activities that regulate *SLC25A37* expression during late-phase erythropoiesis [26]. Further, we checked the expression patterns profiled by the ENCODE consortium for *SLC25A37* on our browser and it showed high tissue specificity to K562 cells (Additional file 1: Figure S1).

### Discovering high-resolution promoter-enhancer interactions using Capture Hi-C and DHS-linkage

**Fig. 3:**
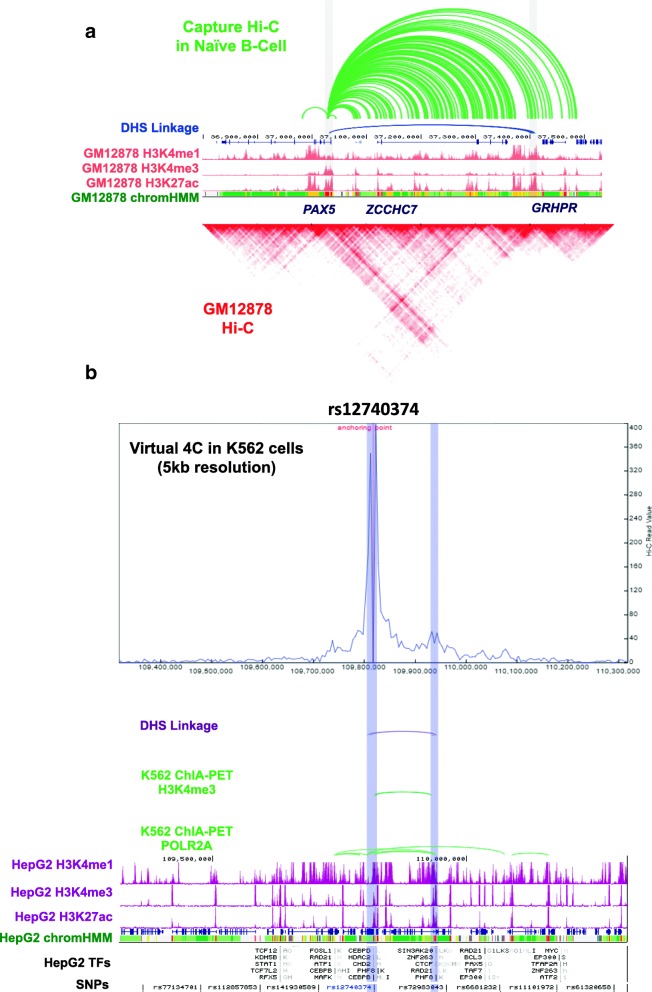
Linking distal regulatory elements and SNPs with their target genes with the 3D Genome browser. **a** Capture Hi-C data in naïve B cells showing potential interactions (green curve lines) with PAX5 promoter region. The Capture Hi-C interactions are consistent with patterns from the 5-kb resolution Hi-C data in GM12878 cells. **b** Using virtual 4C, DHS-linkage, and ChIA-PET data to hypothesize the target gene for non-coding variant rs12740374. Based on the annotation by chromHMM in HepG2, this SNP is located at a putative enhancer region (orange). According to virtual 4C data, there is a potential interaction between this enhancer and the SORT1 promoter. This linkage is also supported by DHS-linkage, as well as by the H3K4me3 and POL2A ChIA-PET data in K562 cell line

While Hi-C data provides a viable way to suggest promoter-enhancer pairing, most of the current published Hi-C maps are at 10–40-kb resolution and therefore are not optimal for uncovering enhancer-promoter interactions. Sequence capture- or pull-down-based methods, such as Capture Hi-C or ChIA-PET, generally have higher resolution and therefore are more effective in identifying chromatin interactions between gene and their *cis*-regulatory elements. In Fig. 3a, we give an example of Capture Hi-C [27], which seeks long-range interactions that involve selected elements of interests captured with pre-determined sequences (in this case, promoters). Capture Hi-C identified chromatin loops are presented as the green arcs (top track in Fig. 3a). The center of the track is the capture sequence in this region, which is the *PAX*-5 gene promoter. We observed that the promoter interacts highly with the nearby regions and most of the interacting regions are enriched for strong enhancer marks (H3K4me1 and H3K27ac).

To further examine the predicted promoter-enhancer linkages, we also explored the linkage data by DNase I hypersensitive sites (DHS) in this region (blue curve line, second track in Fig. 3a), which represents another method of linking distal regulatory element with their target genes. It works by computing Pearson correlation coefficients between the gene proximal and distal DHS pairs across more than 100 ENCODE cell types, and only the pairs with PCC > 0.7 and within 500 kb are kept as the linked pairs [28]. In the example shown in Fig. 3a, we observed several interactions involving the promoter of the *PAX-5* gene and a potential enhancer (marked by both H3K4me1 and H3K27ac signals) downstream of the *ZCCHC7* gene in the naïve B cell Capture Hi-C dataset [27]. One region marked by enhancer-associated histone modifications has indeed been previously determined to be an enhancer for *PAX5*, and its disruption leads to leukemogenesis [29]. By integrating multiple lines of evidence, our browser provides a valuable resource for investigators to generate hypotheses connecting distal non-coding regulatory elements and their target genes.

### Investigating potential target genes for non-coding genetic variants

Resolutions at loci-specific levels also hold significance in the discovery of the functions of non-coding genetic variants, such as single nucleotide polymorphisms (SNPs), which may disrupt transcription factor (TF) binding sites of *cis*-regulatory elements. In this section, we will first demonstrate how to use virtual 4C mode for such analyses. The 4C (circular chromosomal conformation capture [30, 31]) experiment is a chromatin ligation-based method that measures *one-*versus*-many* interactions in the genome, that is, the interaction frequencies between a “bait” locus and any other loci. Its data is plotted as a line histogram, where the center is the “bait” region and any peak signals in distal regions indicate the frequency of chromatin interaction events. In our browser, we use the queried region (gene name or SNP) as the bait and extract Hi-C data centered on the bait region, hence, *virtual* 4C. To bolster the power of the virtual 4C plot, our browser also supplements ChIA-PET and DHS-linkage data. In Fig. 3b, we queried the SNP rs12740374 in the virtual 4C mode. This SNP has been associated with high plasma low-density lipoprotein cholesterol (LDL-C) [32], which could lead to coronary artery disease and myocardial infarction. We plotted virtual 4C and ChIA-PET data from K562 in this region, as high-resolution Hi-C and ChIA-PET data are only available for K562, but not for hepatic cell lines. Since LDLs are processed by the liver, we examined the histone modifications in the Hep2G cell line and found rs12740374 is located within a candidate enhancer region as marked by H3K27ac signals. Hence, virtual 4C, ChIA-PET, and DHS-linkage all support a putative interaction between the enhancer harboring this SNP and the promoter region of *SORT1*. Further, it has been shown that the rs12740374 minor allele creates a C/EBPα-binding site which enhances *SORT1* expression leading to decreased LDL-C levels, thus suggesting that the minor allele confers a gain-of-function effect [33]. Still, despite the unusual conclusions reached by the study—as most minor alleles are usually loss-of-function—the virtual 4C mode of our 3D Genome Browser could aid in the hypothesis generation of not only the *cis*-regulatory elements and their putative target genes but also the effects of non-coding variants.

### Exploring conservation of chromatin structure across species

**Fig. 4:**
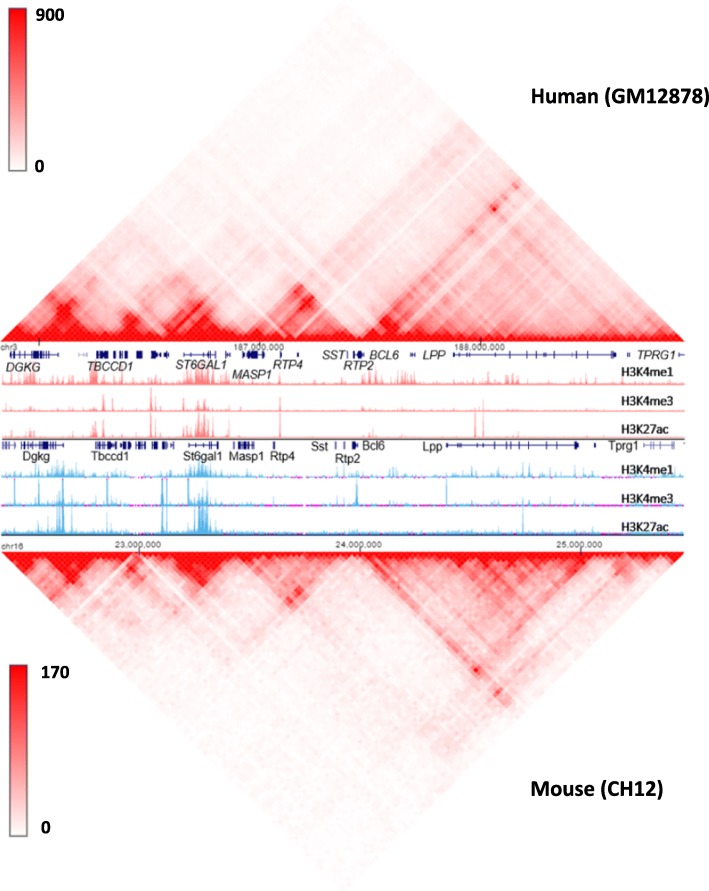
Using the 3D Genome Browser to explore conserved chromatin structure across human and mouse. The similarity between human GM12878 Hi-C data and mouse CH12 Hi-C data at the region surrounding the *BCL6*/Bcl6 gene indicates an evolutionary conservation event of the chromatin structure between the two species

Studying the evolutionary conservation of TADs could lead to a deeper understanding of their functional significance. The compare Hi-C mode of the 3D Genome Browser facilitates this endeavor by stacking Hi-C heatmaps from homologous regions of different species for visual contrast. In this mode, we observed the conservation of TADs and the genes near or at the TAD boundaries between human and mouse in their homologous region surrounding the *BCL-6*/Bcl-6 genes (Fig. 4), suggesting the chromatin structure may play a conserved role in the regulation of this proto-oncogene. This mode could be helpful for users to observe conserved or dynamic Hi-C interactions from different tissue/cell types.

### Uncovering structural variations in cancer genomes

**Fig. 5:**
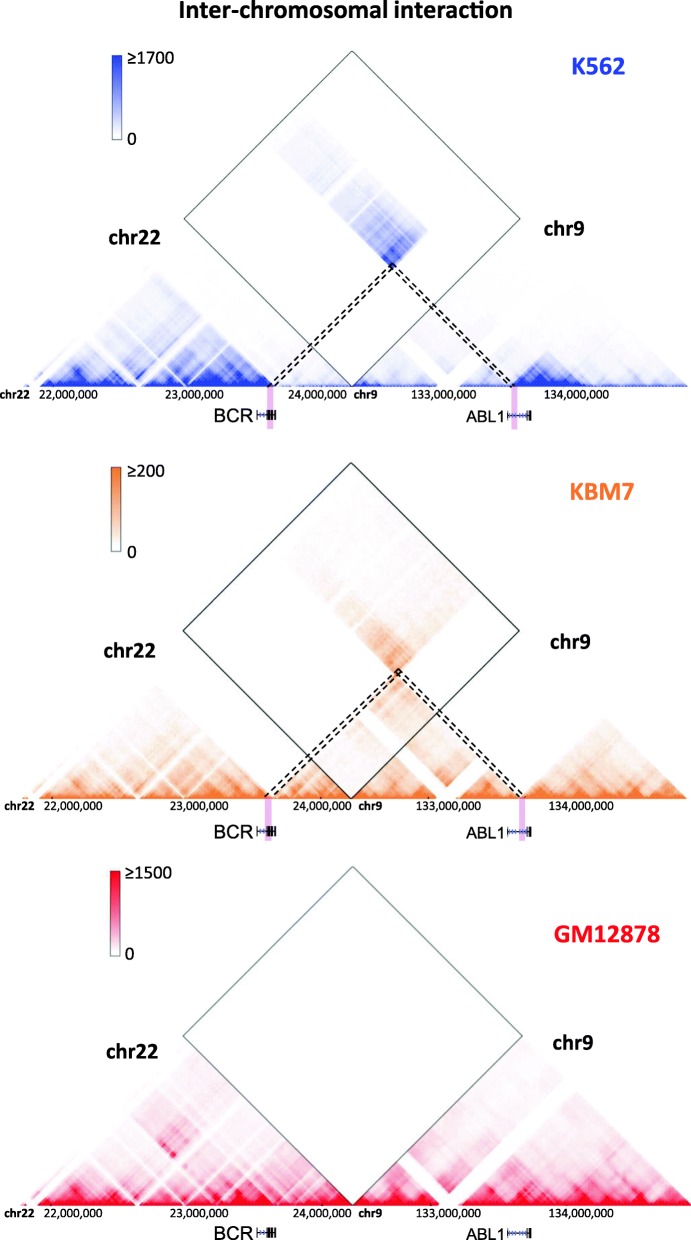
Using the inter-chromosomal interaction mode of the 3D Genome Browser to discover structural variations in cancer cells. An inter-chromosomal translocation event (*BCR-ABL* fusion) in K562 and KBM7 CML cell lines appears as “inter-chromosomal interactions” on Hi-C maps. Such aberrant patterns are frequently observed in Hi-C maps in cancer cells, because the cancer genome is not available and Hi-C reads are mapped to the reference genome. We also noted that this translocation is reciprocal in KBM7 but not in K562 cells and that the breakpoint in ABL is different in the two cell lines. Such inter-chromosomal interactions are not observed in the karyotypically normal GM12878 cells

It has been shown recently that Hi-C data cannot only be used to detect chromatin interactions, but also may be used to denote structural variations [34–39]. Certain structural variations, such as deletions, insertions, inversions and translocations, establish signature patterns have been observed in Hi-C heatmaps. A striking structural variation is shown in Fig. 5 through the inter-chromosomal heatmap mode: we confirmed the oncogenic BCR-ABL inter-chromosomal translocations in two chronic myelogenous leukemia (CML) cell lines, K562 and KBM7. Such inter-chromosomal interactions are not observed in the karyotypically normal GM12878 cell line. We also noted that this translocation is reciprocal in KBM7 but not in K562 cells and that the breakpoint in ABL is different in the two cell lines. In addition, with the browser’s compare Hi-C mode, the users could contrast the similarities and differences of chromosomal structure between distinct cells/tissues or even different species. Comparing the cancer cell line K562 to the normal cell line KBM7, we noted deletions specific to K562, one of which encompasses the tumor suppressor genes *CDKN2A* and *CDKN2B* (Additional file 1: Figure S2), as previously confirmed [40].

### New binary Hi-C data format allows faster data retrieval and visualizing users’ own Hi-C datasets

The 3D Browser supports a variety of features that allow users to browse unpublished data. First, our browser encourages integration with customized UCSC or WashU Epigenome browser sessions, wherein the users could add or modify existing tracks or upload their own genomic/epigenomic data. For example, to view a customized UCSC session, a user would only be required to enter the UCSC session URL. More importantly, the users could view their own Hi-C data by converting the contact matrices into a novel, indexed binary file format called *B*inary *U*pper *T*riangu*L*ar *M*at*R*ix (BUTLR file) developed by us. By hosting the BUTLR file on any HTTP-supported server and providing the URL to the 3D Genome Browser, a user can take full advantage of the features of our browser, without having to upload their Hi-C data since the browser would only query the selected region through binary indexing, rather than searching through the entire matrix. This capability is similar to the bigWig/bigBed mechanism invented by us and UCSC [41].

Additionally, BUTLR format dramatically reduces the file size of high-resolution Hi-C data not only through the binarization but also through the omission of redundant values (Additional file 1: Figure S3a; Additional file 2). The BUTLR file encodes an entire genome-wide chromatin interactions data into a binary, indexed format. While 1-kb resolution hg19 intra-chromosomal Hi-C contact matrices in the tab-delimited format require almost 1 TB, the BUTLR format of those same matrices would only take 11 GB (Additional file 1: Figure S3b). More importantly, the binary file format also greatly improves the query speed: using pre-loaded Hi-C datasets, the 3D browser generally returns the query results as a heatmap in a matter of seconds. We also want to note that our browser is designed as query-based to maximize its usability, and as a result, it excels at exploring locus of interest and gene-element relationship, but can be a little less dynamic than other tools when navigating Hi-C matrix for larger genomic regions.

## Conclusion

In summary, we developed an interactive 3D Genome Browser that is defined by simple and easy-to-navigate graphical user interface, fast query-response time, and a comprehensive collection of publicly available chromatin interaction datasets. As our browser simultaneously displays the 3D chromatin interactions, functional (epi)genomic annotations, and disease/trait-associated SNPs, we provide an invaluable online tool for investigators from all over the world for the study of 3D genome organization and its functional implications in mammalian gene regulation.

## Methods

### Backend and user interface

The 3D Genome Browser is supported by the LAMP (Linux, Apache, MySQL, PHP) stack web service on the backend. At the user-interface level, the browser depends on HTML5 and JavaScript and its libraries JQuery and D3.js. All displays are rendered on HTML5 Canvas or Inline SVG.

### In-house Hi-C data processing pipeline

We followed the pipeline in Dixon et al. [22] for Hi-C data processing. Briefly, raw fastq files were aligned to human reference genome GRCh38 with BWA aligner (0.7.15-r1140). Only uniquely mapped reads and properly paired reads on the same chromosome are retained. The genome is binned at different resolution (e.g., 40 kb and 10 kb) to generate Hi-C matrix. Paired reads were considered to be chromatin interactions connecting two bins. ICE (iterative correction and eigenvector decomposition) normalization was done using the “iced” Python package.

### User query submission

The user may provide genomic coordinates or genome features such as gene symbols, RefSeq ID, Ensembl ID, or SNP rsid as queries for all modes of the 3D Genome Browser.

### External genome browser integration and alignment

For the UCSC Genome Browser, we embed its sessions with the iframe and we align its content with our tracks by manipulating the scroll bars of the div HTML element containing the iframe. The WashU Epigenome Browser provides a JavaScript function for seamless integration into our browser. For both external browsers, it is possible for the user to embed a user-defined session consisting of user-selected tracks and options by providing the session URL to the 3D Genome Browser.

### Determining homologous regions

For the compare Hi-C mode, we determine the homologous regions between two species by querying for homologous genes from the NCBI’s HomoloGene database [42] as well as utilizing known inter-species chains [43].

### BUTLR format

The BUTLR file encodes an entire genome-wide chromatin interactions data into a binary, indexed format. To compress the original contact matrices, BUTLR only stores the nonzero values of the upper triangular matrices of the intra-chromosomal data and the *n × m*, where *n* and *m* are the number of interrogated loci and where *n* < *m* of the inter-chromosomal data. The locations of each chromosome or chromosome-pair matrix, row indices of each matrix, and column indices of nonzero values along with nonzero values are binarized and indexed within the BUTLR file structure. Perl scripts that encode and decode BUTLR files are available at http://github.com/yuelab/BUTLRTools. All the Hi-C matrices in this manuscript are converted to BUTLR file format for visualization [5, 8, 19, 20, 22–24, 44-54].

## Additional files

Additional file 1: **Figure S1.** Gene expression of *SCL25A37* across 109 issues. **Figure S2.** Using the 3D Genome Browser to determine intra-chromosomal structural variations. **Figure S3.** Design and performance of the BUTLR file format. **Table S1.** List of Hi-C datasets hosted by the 3D Genome Browser.** Table S2.** List of ChIA-PET, Capture Hi-C, PLAC-Seq and HiChIP datasets. **Table S3.** List of GAM, DNase Hi-C, and SPRITE datasets. (PDF 1295 kb)

Additional file 2: Review history. (DOCX 1354 kb)
