# Supplementary material for: The 3D Genome Browser: a web-based browser for visualizing 3D genome organization and long-range chromatin interactions
Source: Genome Biol. 2018 Oct 4;19:151. doi: 10.1186/s13059-018-1519-9 (PMC6172833; doi:10.1186/s13059-018-1519-9)
Supplement: Supplementary file 1 — Figure S1. Gene expression of SCL25A37 across 109 issues. Figure S2. Using the 3D Genome Browser to determine intra-chromosomal structural variations. Figure S3. Design and performance of the BUTLR file format. Table S1. List of Hi-C datasets hosted by the 3D Genome Browser. Table S2. List of ChIA-PET, Capture Hi-C, PLAC-Seq and HiChIP datasets. Table S3. List of GAM, DNase Hi-C, and SPRITE datasets. (PDF 1295 kb) [file 13059_2018_1519_MOESM1_ESM.pdf]

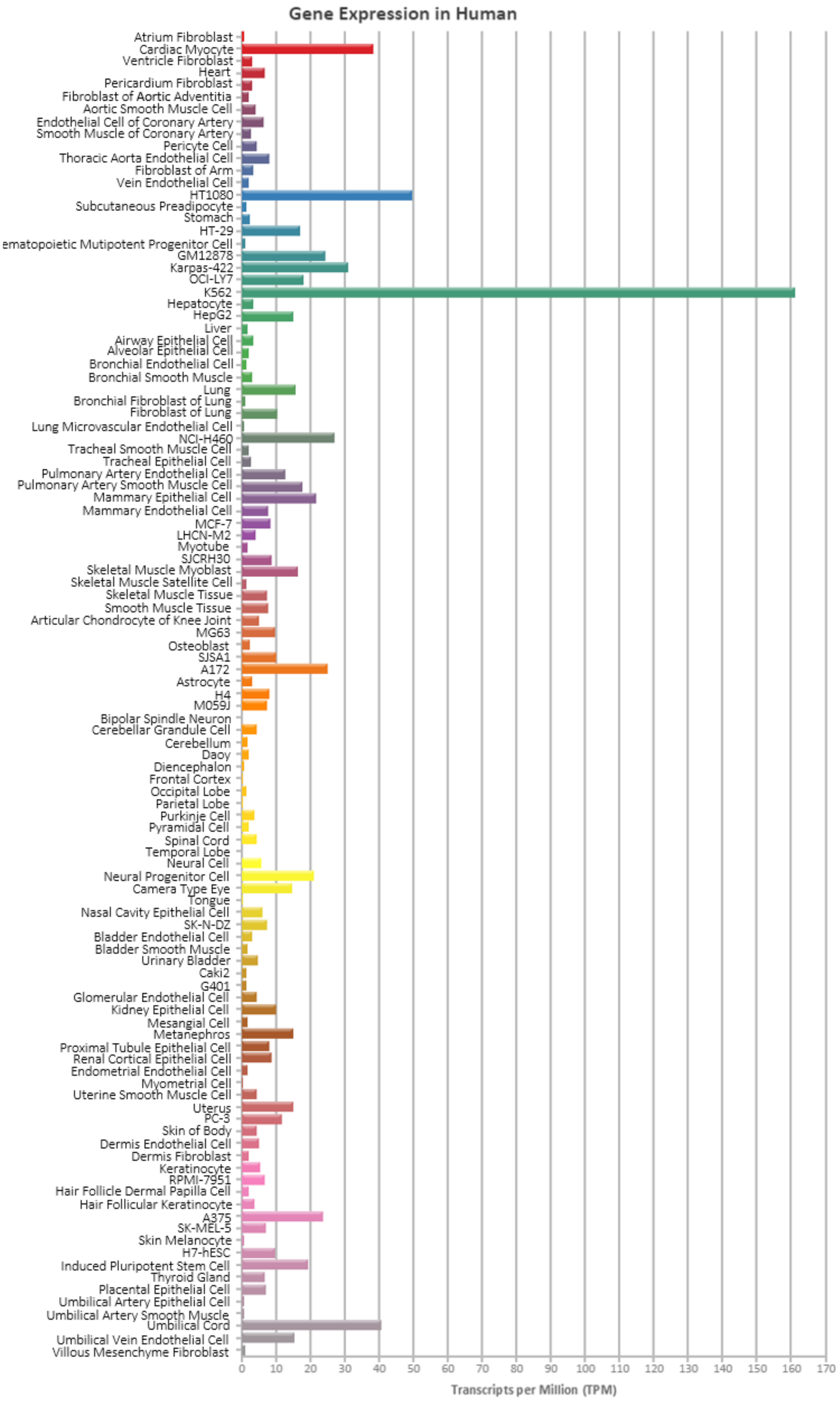

Figure S1. Gene expression of *SCL25A37* across 109 tissues. *SLC25A37* expression in K562 is more than three times as much compared to other 108 tissues.

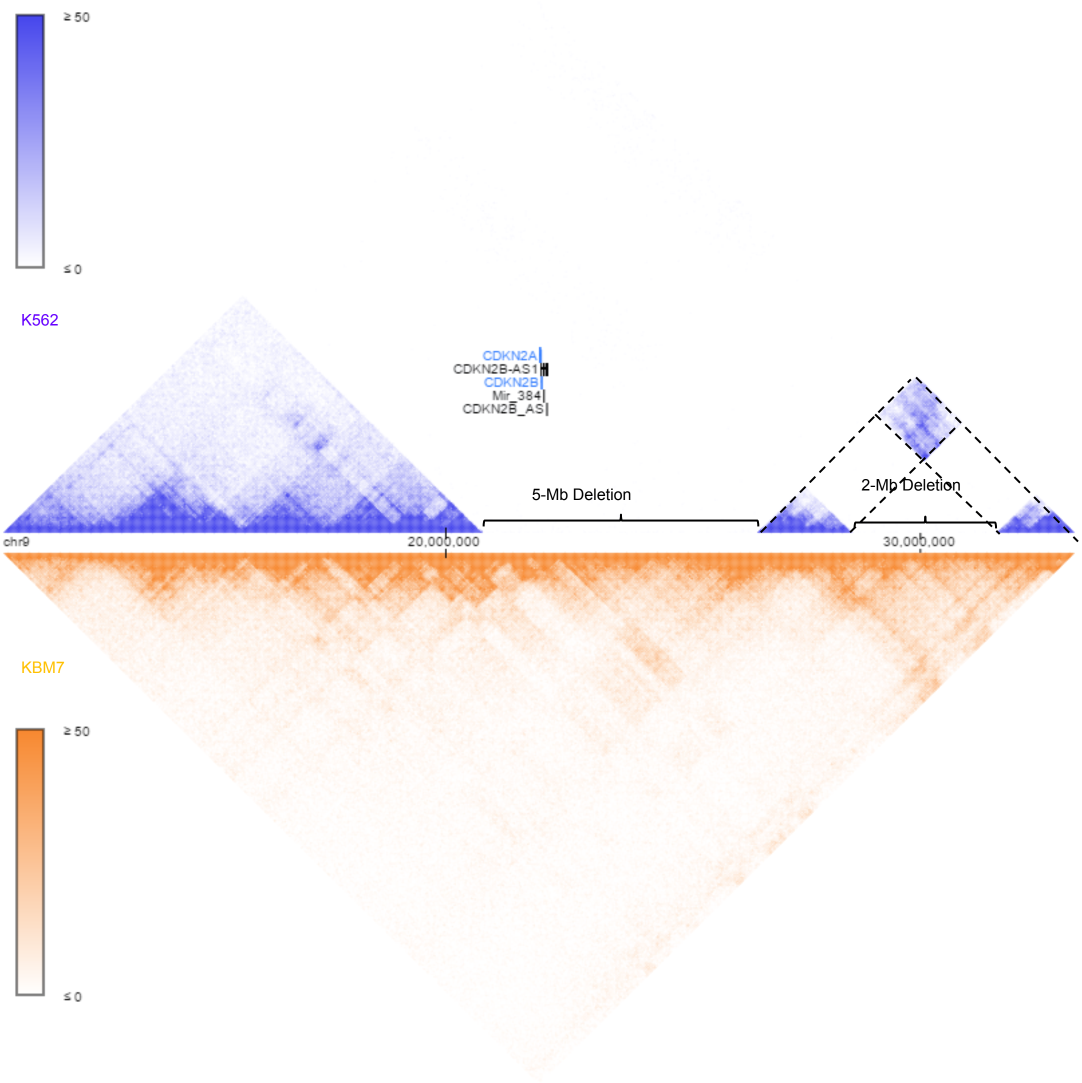

Figure S2. Using the 3D Genome Browser to determine intra-chromosomal structural variations. There is a large deletion encompassing CDKN2A and CDKN2B region, specific to K562 on chromosome 9.

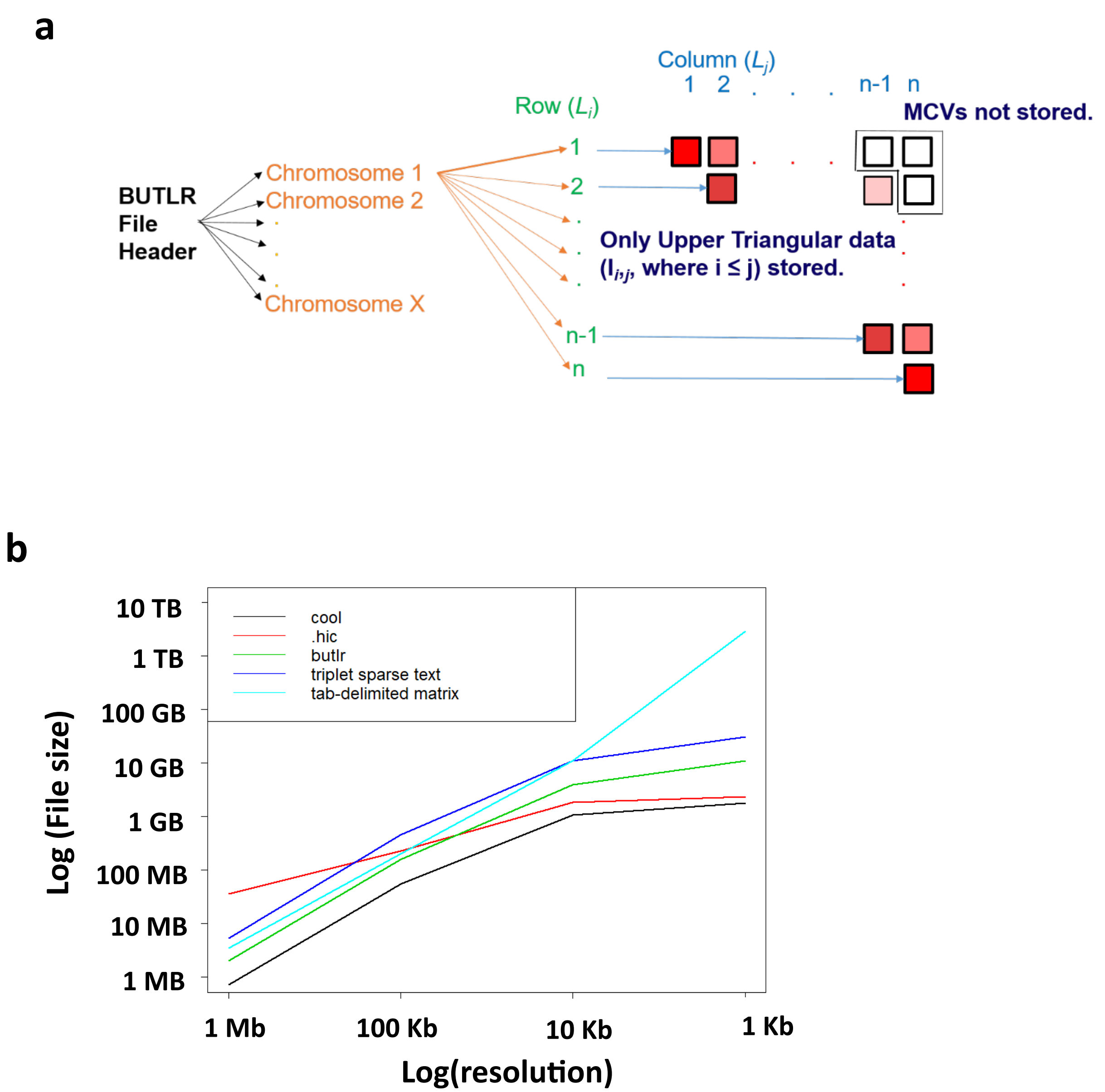

Figure S3. Design and performance of the BUTLR file format. **a** The BUTLR format encodes the chromosome or chromosome pairs (inter-chromosomal), row and column information as a series of pointers (represented by arrows). For purposes of compression, the most common values (MCVs) and their column locations are omitted from storage. **b** Comparing the file sizes of Hi-C matrices by BUTLR, tab-delimited matrix, .hic, cool, and triplet sparse text formats.

Table S1. List of Hi-C datasets hosted by the 3D Genome Browser.

Species	Assembly	Tissue	Resolution	Source
Human	hg19, hg38	GM12878	1-kb, 5-kb, 10-kb, 25-kb (hg19)<br>10-kb, 25-kb, 40-kb (hg38)	Rao et al., 2014 [24]
		K562	5-kb, 10-kb, 25-kb (hg19)<br>10-kb, 25-kb, 40-kb (hg38)	
		IMR90		
		HMEC		
		HUVEC		
		NHEK		
		KBM7		
Mouse	mm9	CH12		
Human	hg19	GM12878	40-kb	Dixon et al., 2012 [5]
	hg19, hg18	IMR90		
Mouse	mm9	ESC	40-kb	Dixon et al., 2012 [5]
	mm9	Cortex		
Human	hg38	H1-ESC	10-kb, 25-kb, 40-kb	Jin et al., 2013 [44]
		IMR90		
		IMR90, TNF-α Treated		
		IMR90, Flavopiridol Treated		
Human	hg18, hg19, hg38	H1-ESC	40-kb (hg18, hg19)<br>10-kb, 25-kb, 40-kb (hg38)	Dixon et al., 2015 [22]
		H1-MES		
		H1-MSC		
		H1-NPC		
		H1-TRO		
Human	hg19, hg38	Aorta	40-kb (hg19)<br>10-kb, 25-kb, 40-kb (hg38)	Leung et al., 2015 [45]
		Left Ventricle		
		Liver		
		Thymus		
Human	hg19, hg38	Adrenal Gland	40-kb (hg19)<br>10-kb, 25-kb, 40-kb (hg38)	Schmitt et al., 2016 [23]
		Bladder		
		Dorsolateral prefrontal cortex		
		Hippocampus		
		Lung		
		Ovary		
		Pancreas		
		Psoas Muscle		
		Right Ventricle		
		Spleen		
Human	hg19, hg38	A549	40-kb (hg19)<br>10-kb, 25-kb, 40-kb (hg38)	ENCODE3 (lab: Dekker)
		Caki2		
		G401		
		LNCaP		
		NCIH460		
		PANCI		
		RPMI7951		
		SJCRH30		
		SKMEL5		
		SKNDZ		
		SKNMC		
Human	hg19	Fetal Brain (Cortical Plate)	10-kb	Won et al., 2016 [46]
		Fetal Brain (Germinal Zone)		
Mouse	mm9	NPC	40-kb	Fraser Laboratory
		Neuron		
Mouse	mm9	Th1	40-kb	Nagano et al., 2015 [47]
Human	hg18	GM06990	1-Mb	Lieberman-Aiden et al., 2009 [8]
	hg38	GM12878	10-kb, 25-kb, 40-kb	
Mouse	Mm10	F123_ES	40-kb	Hardison Laboratory

		G1E-ER4		
		HPC7		
**Human**	hg38	Primary_Epidermal_Keratinocyte at day0	10-kb, 25-kb, 40-kb	Rubin et al., 2017 [48]
		Primary_Epidermal_Keratinocyte at day3		
		Primary_Epidermal_Keratinocyte at day6		
**Human**	hg38	HCT-116_RAD21-mAC	10-kb, 25-kb, 40-kb	Rao et al., 2017 [49]
		HCT-116_RAD21-mAC with auxin treatment		
**Human**	hg38	THP-1	10-kb, 25-kb, 40-kb	Phanstiel et al, 2017 [50]
		THP-1 with PMA treatment		
**Mouse**	mm10	Neuron	10-kb, 25-kb, 40-kb	Jiang et al., 2017 [51]
		Neuron_Setdb1_KnockOut		
**Mouse**	mm10	ESC	10-kb, 25-kb, 40-kb	Bonev et al., 2017 [52]
		NPC		
		Cortical Neuron		
**Mouse**	mm10	Myoblast	10-kb, 25-kb, 40-kb	Doynova et al., 2017 [53]

Table S2. List of ChIA-PET, Capture Hi-C, PLAC-Seq and HiChIP datasets.

Species	Method	Tissue	Enrichment Target	Source
Human	ChIA-PET	K562	CTCF	ENCODE2 (lab: Ruan)
			H3K4me1	ENCODE3 (lab: Snyder)
			H3K4me2	ENCODE3 (lab: Snyder)
			H3K4me3	ENCODE3 (lab: Snyder)
			H3K27ac	ENCODE3 (lab: Snyder)
			POLR2A	ENCODE2 (lab: Ruan)
			RAD21	ENCODE3 (lab: Snyder)
		HCT116	POLR2A	ENCODE2 (lab: Ruan)
		MCF-7	CTCF	ENCODE2 (lab: Ruan)
			ESR1	ENCODE2 (lab: Ruan)
			POLR2A	ENCODE2 (lab: Ruan)
		GM12878	RAD21	ENCODE3 (lab: Snyder)
		NB4	POLR2A	ENCODE2 (lab: Ruan)
		HeLa-S3	POLR2A	ENCODE2 (lab: Ruan)
Human	HiChIP	GM12878	Cohesin	Mumbach et al., 2016 [13]
Mouse		ESC	Cohesin	
Mouse	PLAC-Seq	ESC	H3K4me3	Fang et al., 2016 [12]
			H3K27ac	
			POL2	
Human	Capture Hi-C	B Naïve	Promoter	Javierre et al., 2016 [27]
		B Total		
		CD4 Activated		
		CD4 Naïve		
		CD4 Total		
		CD4 Nonactivated		
		CD8 Naïve		
		CD8 Total		
		Endothelial Precursors		
		Erythroblasts		
		Fetal Thymus		
		Macrophages M0		
		Macrophages M1		

		Macrophages M2		
		Megakaryocytes		
		Monocytes		
		Neutrophils		
		CD34		Mifsud et al., 2015 [11]
		GM12878		

Table S3. List of GAM, DNase Hi-C, and SPRITE datasets.

Species	Assembly	Tissue	Resolution	Source
**Mouse**	mm9	ESC	1-Mb, 30-kb	Beagrie et al., 2018 [19]
**Human**	hg19	K562	1-Mb	Ma et al., 2015 [54]
		H1-ESC		
**Human**	hg19	GM12878	25-kb, 50kb, 250kb, 1-Mb	Quinodoz et al., 2018 [20]
**Mouse**	mm9	ESC	20-kb, 40-kb, 100-kb, 200-kb, 400-kb, 1-Mb	
