# Supplementary material for: The 3D Genome Browser: a web-based browser for visualizing 3D genome organization and long-range chromatin interactions
Source: Genome Biol. 2018 Oct 4;19:151. doi: 10.1186/s13059-018-1519-9 (PMC6172833; doi:10.1186/s13059-018-1519-9)
Supplement: Supplementary file 2 — Review history. (DOCX 1354 kb) [file 13059_2018_1519_MOESM2_ESM.docx]

Review History

**First round of review**

**Reviewer 1**

**Are the methods appropriate to the aims of the study, are they well described, and are necessary controls included?**
Yes

**Are the conclusions adequately supported by the data shown?**

Yes

**Are sufficient details provided to allow replication and comparison with related analyses that may have been performed?**

Yes

**Does the method perform better than existing methods (as demonstrated by direct comparison with available methods)?**

Yes

**Is the method likely to be of broad utility? Is any software component easy to install and use?**

Yes.

**Is the paper of broad interest to others in the field, or of outstanding interest to a broad audience of biologists?**

Yes: Should the authors continue to improve the 3D genome browser, as suggested, this web tool can be really useful to experimentalists in various research fields.

Comments to author:

In the manuscript GBIO-D-17-01760, Wang et al present the development of a web tool, the 3D genome browser, for visualizing 3D genome organization and long-range chromatin interactions. This portable and interactive browser, which features six modes including Hi-C heatmap, virtual 4C, ChIA-PET/HiChIP/PLAC-seq, Capture Hi-C and interchromosomal interactions, allows users to conveniently explore chromatin interactome data tailored toward their own needs, from both published (over 300 datasets) and their own chromatin interaction data, by providing convenient zoom and traverse functions in real time and supporting queries by gene name, genomic loci, or SNP ID.

With the emerging impacts of the 3D genome on various biological processes and human diseases, and with reference chromatin interaction maps being generated in increasing cell/tissue types, use-friendly web tools, which enable general experimentalists (i.e., those without much computational skills) to navigate 3D genome maps along with other genomic and epigenomic information, are in great need. In this regard, the 3D genome browser, which simultaneously displays chromatin interactions, functional genomic annotations, and disease/trait-associated SNPs, indeed provides a convenient and practical online tool. However, as listed below, the browser can be improved in several aspects to benefit general users.

Specific concerns:

1)	One of the noticeable limitations of the current implementation of the browser is the lack of result export function. In all the six modes, only part of the results (the image or part of the image but not together with the aligned tracts) can be saved/downloaded.

2)	The current implementation of the browser is specific to Hi-C datasets generated by the traditional restriction enzyme-based Hi-C methods. However, there are now increasing chromatin interaction datasets generated by either other Hi-C variants (e.g., DNase Hi-C, Micro-C) or non-Hi-C methods (e.g., GAM, Spritie, and TSA-seq). It will benefit the research community if the authors can also host these datasets in the browser.

3)	One of the features of the browser is its large number of datasets, will the Yue lab plan to keep uploading new public available Hi-C datasets? How will the authors ensure the long-term availability/stability of this online server?

4)	There are now about a dozen of online tools available for visualizing and analyzing Hi-C datasets. A recent publication (Tang et al, 2017, Bioinformatics) compared these tools and indicated that the Delta browser has more useful features than all the other tools including the 3D genome browser. Do the authors agree with this result? Why?

5)	In the ChIA-pet & similar and capture Hi-C modes, are the visualized interactions normalized or not (ie, raw data)? Is there any statistical cut off, i.e., only statistically significant interactions will be visualized?

6)	All the abbreviations in the paper need to be defined.

7)	In general, the quality of the main figures can be improved.

**Reviewer 2:**

**Are the methods appropriate to the aims of the study, are they well described, and are necessary controls included?**
Yes

**Are the conclusions adequately supported by the data shown?**

Yes

**Are sufficient details provided to allow replication and comparison with related analyses that may have been performed?**

Yes

**Does the method perform better than existing methods (as demonstrated by direct comparison with available methods)?**

No: "Performing better" is difficult to estimate here. This tool offers new possibility to visualize Hi-C data

**Is the method likely to be of broad utility? Is any software component easy to install and use?**

Yes.

**Is the paper of broad interest to others in the field, or of outstanding interest to a broad audience of biologists?**

Yes: This could be an interesting ressources for people interested in chromosome conformation data

Comments to author:

The authors present here the 3D Genome Browser, a web-based interface allowing Hi-C-based data visualization. Compared to existing solution, the 3D Genome Browser offers alternative views such the virtual 4C or the capture-C representation. It also offers access to many datasets, as well as UCSC or WashU genome browser which is extremely useful for biologists who already have their tracks on such browser. Therefore, I'm sure that this tool could be useful for anyone interested in exploring chromosome conformation data.

Regarding the manuscript, I'm listing here a couple of points in order to improve it.

- Line 69 - The authors mention that the 3D Genome Browser is portable ? What does it mean ? To me, the term "portable" refers to something that can be installed on different environment … which is not the case here, as the tool is only available on-line.

- Line 91 - Data have been re-processed by an in-house data processing pipeline. Is there any description of this pipeline somewhere ?

- Line 98 - " (…) We also collected the open chromatin interaction data from (..)". I think this part refers to Dnase I data. Please, do not use the word "interaction" here.

- Line 104 - typo "these regionS"

- Line 150 - Please introduce what is DHS

- Line 205 - how can you state that the translocation is balanced in KBM7 and unbalanced in K562 ? Do you clear evidence of that ?

- Sup Fig 3 - what does tab-delimited matrix means ? Could you add here comparison with other Hi-C data compression format such as the .hic format (for Juicebox), the cooler format (for HiGlass), or the text triplet sparse format (proposed by HiC-pro) ?

- Overall the paper is well written, but some paragraphs need careful review in terms of language and structure.

In addition, I tested the interface, and did not have any trouble playing with already published data. I'm also adding a few comments about the tool itself.

- The 3D Genome Browser has some limitations when we want to look at large genomic regions (as complete chromosome for instance). This should be mentioned/discussed somewhere, as one of its limitations compared to other solution.

- It is also less dynamic than other tools such as Juicebox and HiGlass, but is maybe more dedicated to the exploration of single locus of interest. This should also be discussed.

- All data are not always available. It would be great to have all normalization/all resolutions maps for all dataset. I did not tested all the dataset, but I only found normalized (iced) data for Human A549 …

- In the same way, I also looked at Mouse Neuron cells, but did not find TADs information ? If having all information for all samples is not possible, a table which summarizes which data is available for which dataset could be useful.

**Authors’ response to reviewers:**

Summary of Changes:

First of all, we want to thank the reviewers for the insightful suggestions. We were delighted to see the overall positive comments from both reviewers. Below we summarize the major changes we introduced to our system according to their suggestions:

1. Export function: we have added export function for all the pages. Now our users can conveniently download both the chromatin interaction images and the aligned genome browser tracks from our website.

2. Visualizing large genomic region: We have rewritten our code so that now the users can visualize large genomic regions at whole chromosome levels.

3. Predicting Topologically Associating Domains (TADs): we have predicted TADs in all the Hi-C datasets in our browser using the same pipeline that we previously designed in Dixon et al. Nature 2012. The new TADs are available for both visualization and download from our website.

4. Normalizing Hi-C data matrix: we have performed ICE (iterative correction and eigenvector decomposition) normalization to all the Hi-C datasets in our browser, and therefore users can explore the bias-corrected Hi-C matrix.

We want to note that our revised manuscript represents one of the most comphrehensive resource to date that provides TAD prediction and normalized Hi-C data. Coupled with the newly added export functions and easy-to-use navigation system, we believe the 3D genome browser will continue to be one of the most used tools to explore chromatin interaction data for the greater scientific community.

Reviewers' Comments:

Reviewer #1

Specific concerns:

1) One of the noticeable limitations of the current implementation of the browser is the lack of result export function. In all the six modes, only part of the results (the image or part of the image but not together with the aligned tracts) can be saved/downloaded. We thank the reviewer for this excellent suggestion. Now, we have added the export function for all the modes and an example screenshot is listed below. Specifically, we also added a function where the users can also directly export the “aligned tracts” from UCSC browser in the same region. Please note that due to technical challenge, we can only generate two separate PDF files (one for chromatin interaction data and one for UCSC tracks), but they cover the exact same region and can be aligned directly by other software such as Powerpoint or Adobe Illustrator.

**Response. Figure 1. A screenshot from the 3D genome browser with the**

**newly added export function.** There are two buttons for the export function,

“Download Heatmap” and “Download UCSC.” The two pdf files contains the Hi-C

data and the UCSC genome browser tracks for the same region.

**2) The current implementation of the browser is specific to Hi-C datasets generated by the traditional restriction enzyme-based Hi-C methods. However, there are now increasing chromatin interaction datasets generated by either other Hi-C variants (e.g.,**

**DNase Hi-C, Micro-C) or non-Hi-C methods (e.g., GAM, Spritie, and TSA-seq). It will benefit the research community if the authors can also host these datasets in the browser.**

This is another excellent point. Following the reviewer’s suggestion, we have added the GAM, SPRITE, DNase Hi-C datasets in our browser on the orginal Hi-C page. The details of the added datasets are listed below:

1. GAM data for mouse embryonic stem cells is from Beagrie *et al.* 2017 (GEO:

GSE64881).

2. Two DNase Hi-C datasets (human embryonic stem cells and K562) are from Ma *et al.*

2015 (https://noble.gs.washington.edu/proj/dnase-hi-c/).

3. Two SPRITE datasets (human GM12878 and mouse embryonic stem cells) are from

Quinodoz *et al.* Cell 2018 (GEO: GSE114242).

4. At the time of this submission, TSA-seq datasets have not been released yet. However, we will add them to our browser as soon as the data become publicly available.

**3) One of the features of the browser is its large number of datasets, will the Yue lab**

**plan to keep uploading new public available Hi-C datasets? How will the authors ensure**

**the long-term availability/stability of this online server?**

This project is currently supported by Dr. Yue’s NIH grant (R01HG009906, *Visualization,*

*Modeling and Validation of Chromatin Interaction Data*) through December 2022. It is also supported by Dr. Ting Wang and Dr. Yue’s NIH grant (U01CA200060, *The WashU 4DN*

*Network Data Coordination and Integration Center*), whose goal is to build and maintain the 4D

Nucleome Consortium Data Coordination and Integration Center. Therefore, we are financially committed to maintaining and updating this website. More importantly, we want to note that the prototype for this website started as early as in 2012, when we published the work that proposed topologically associating domains (Dixon et al. Nature 2012). In the past six years, we have been vigoriously maintaining and updating both the database and the features for this browser. As shown below, it has formed a large scientific community gradually with more than 60,000 users and 648,475 page views. Therefore, we are fully determined to maintain this invaluable tool for the scientific communicty.

Response Fig. 2. Screenshot of the statistics of users of the 3D Genome Browser (Google analytics).

**4) There are now about a dozen of online tools available for visualizing and analyzing Hi-**

**C datasets. A recent publication (Tang et al, 2017, Bioinformatics) compared these tools**

**and indicated that the Delta browser has more useful features than all the other tools**

**including the 3D genome browser. Do the authors agree with this result? Why?**

https://academic.oup.com/bioinformatics/article/34/8/1409/4747883

http://delta.big.ac.cn/index.jsp

We thank the reviewer for pointing out the Delta browser and we have added proper references to the tools listed in the Tang et al, 2017 paper.

The reviewer is absolutely right that there are several tools available for visualizing the Hi-C data. **We believe each of them has their unique features and can complement each other.**

For example, we agree that Delta browser has many useful features compared with other tools, in particular, the elegant integration of physical view of 3D genome modeling and Hi-C data.

At the same time, we believe our browser has its own unique features that makes it one of the most used tools for exploring chromatin interaction data. The features are listed below:

1. Most comphrehensive chromatin interaction data types: so far we are the only system that hosts Hi-C, Capture Hi-C, ChIA-PET, Hi-ChIP, PLAC-Seq, GAM and SPRITE data.

2. Large number of pre-loaded and processed Hi-C datasets. In total, we have 104 Hi-C datasets in multiple species and assemblies.

3. Virtual 4C and DNase I HSS linkage mode to link enhancers with their target genes;

4. Universally processed Hi-C data, including TAD prediction and normalized Hi-C matrix;

5. Unique binary file format, which greatly increases the query and visualization speed;

6. Inter-chromosomal interactions as a heatmap – this is a great tool for discovering and examining the chromsomal translocation events using Hi-C data.

7. Seamless integration of UCSC and WashU genome browser with the chromatin interactions.

8. Compare Hi-C mode, where Hi-C data has been normalized and shown as a differential

heatmap. This feature is very help for identifying differential chromatin interactions and only available in our browser.

**5) In the ChIA-pet & similar and capture Hi-C modes, are the visualized interactions normalized or not (ie, raw data)? Is there any statistical cut off, i.e., only statistically significant interactions will be visualized?**

We’d like to apologize for not articulating well enough in the first submission. The displayed interations for ChIA-PET and capture Hi-C data are the results reported in the original papers.

We have added clarifications in the manuscript. Below we summarize how these interactions were identified in the original work:

ChIA-PET (Li, G *et al*. Cell. 2012): Paired-end tags were first clustered into self-ligated an inter ligated tags. Inter-ligated tags were used to profile the interactions between two anchor regions. The interaction was then normalized by the tag count in two anchors and the sequencing depth using a hyper-geometric model. The p values were corrected as FDR for multiple hypothesis testing using BH method (Benjamini and Hochberg, 1995). Interactions with

FDR < 0.05 were reported as significant.

Capture Hi-C (Mifsud, B *et al*. Nat, Genet 2015): Significant interactions were detected using

GOTHiC. GOTHiC applies a binomial model followed by BH correction to separate true interactions from background noises. A FDR cutoff of 0.05 was used. To eliminate artifacts, interacting fragment coverage was further required to be within 0.2 to 3-fold of the median coverage of singleton fragments.

**6) All the abbreviations in the paper need to be defined.**

Thanks for the suggestion. We have systematically examined the manuscript and made sure the abbreviations were clarified when they first appear in the manuscript.

**7) In general, the quality of the main figures can be improved.**

Thanks for the suggestion. Now we have added the export function for almost all the pages and we have replaced the panels with high-resolution images.

**Reviewer #2: The authors present here the 3D Genome Browser, a web-based interface allowing Hi-C-based data visualization. Compared to existing solution, the 3D Genome**

**Browser offers alternative views such the virtual 4C or the capture-C representation. It also offers access to many datasets, as well as UCSC or WashU genome browser which is extremely useful for biologists who already have their tracks on such browser.**

**Therefore, I'm sure that this tool could be useful for anyone interested in exploring chromosome conformation data.**

We thank the reviewer for the positive comments.

**Regarding the manuscript, I'm listing here a couple of points in order to improve it.**

**- Line 69 - The authors mention that the 3D Genome Browser is portable ? What does it mean ? To me, the term "portable" refers to something that can be installed on different environment … which is not the case here, as the tool is only available on-line.**

The reviewer is right in pointing out the usage of the word “portable”. Originally, we meant that users can upload their data and explore them using different devices. We have since removed this word to avoid any confusion.

**- Line 91 - Data have been re-processed by an in-house data processing pipeline. Is there any description of this pipeline somewhere ?**

Thanks for the suggesiton. Now we have added the following descriptions in the method section:

“*We followed the pipeline in Dixon et al 2015 for Hi-C data processing. Briefly, raw fastq files were aligned to human reference genome GRCh38 with BWA aligner (0.7.15-r1140). Only uniquely mapped reads and properly paired reads are retained. The genome is binned at different resolution (e.g. 40kb, 10kb, etc.) to generate HiC matrix. Paired reads were considered to be chromatin interactions connecting two bins.. ICE (iterative correction and eigenvector decomposition) normalization was performed using the “iced” Python package.”*

**- Line 98 - " (…) We also collected the open chromatin interaction data from (..)". I think this part refers to Dnase I data. Please, do not use the word "interaction" here.**

We thank the reviewer for the suggestion and have fixed it.

**- Line 104 - typo "these regionS"**

Fixed.

**- Line 150 - Please introduce what is DHS**

We appologize for not explaining the abbreviation in the first submission. DHS stands for DNase

I Hypersensitive Sites and we have now fixed it in the manuscript.

**- Line 205 - how can you state that the translocation is balanced in KBM7 and unbalanced in K562 ? Do you clear evidence of that ?**

We deeply appreciate the reviewer for pointing out this misuse of the word – what we meant is that this is a “reciprocal translocation”. In another manuscript from our group and our collaborators (Dixon et al., Biorxiv, 2017, accepted in Nature Genetics), we designed an algorithm that can use Hi-C data to detect different types of SVs. As shown in the below panel, two chromosomes can be fused to each other by four types of orientation combinations. For example, a 5’3’ translocation means the upstream part from the breakpoint in chr22 is fused with the downstream part from the breakpoint on chr9. In this scenario, we can observe Hi-C signals in the upper quadrant in both KMB7 and K562 (Right upper panel). A 3’5’ translocations means that the downstram part from the breakpoint in chr22 is fused with the upstream part from the breakpoint on chr9. This is reflected on a Hi-C map as the signals in the lower quadrant – we only observed such pattern in KMB7, but not in K562. Therefore, we make the claim that “*this translocation is reciprocal in KBM7, but not in K562 cells*”.

Response Figure 3. Diagram illustrating that Hi-C data can be used to identify different types of structural variations.

**- Sup Fig 3 - what does tab-delimited matrix means ? Could you add here comparison with other Hi-C data compression format such as the .hic format (for Juicebox), the cooler format (for HiGlass), or the text triplet sparse format (proposed by HiC-pro) ?**

The tab-delimited matrix file is essentially just a Hi-C interaction matrix in a text file. Each row or column is a bin on the genome and the columns are separated by tabs. The text triplet sparse format is also a text file only storing the upper or lower triangular non-zeros values. Its first two columns are the coordinates of two interaction regions, and third column is the contact frequency.

Following the reviewer’s suggestion, we added the comparison of file sizes with .hic format, cooler fomat, and the triplet sparse format. To do a fair comparison, we started from the same dataset which contains all the intrachromosomal interactions for GM12878 from Rao et al 2014

(GEO: GSE63525). The downloaded data is already in text triplet sparse format. Then we converted it to tab-delimited matrix and Butlr format using script from our browser (http://promoter.bx.psu.edu/hi-c/butlr.html). To generate .hic and cooler format, we first converted triplet sparse format to 4DN pairs format (<https://github.com/4dndcic/> pairix/blob/master/pairs_format_specification.md), then we used the Juicer tools (version 1.8.9) to generate the .hic format, and Cooler python package (version 0.7.10) to generate the cooler format from 4DN pair format. We have updated the Supplementary Fig. 3 with the new results.

Supplementary Figure 3b. Comparing the file sizes of Hi-C matrices in BUTLR, tab-delimited matrix, hic, cool, and triplet sparse text formats.

**- Overall the paper is well written, but some paragraphs need careful review in terms of**

**language and structure.**

We thank the reviewer for this comment and have closely gone over the paper multiple times to improve the clarity of the manuscript.

**In addition, I tested the interface, and did not have any trouble playing with already published data. I'm also adding a few comments about the tool itself.**

**- The 3D Genome Browser has some limitations when we want to look at large genomic regions (as complete chromosome for instance). This should be mentioned/discussed somewhere, as one of its limitations compared to other solution.**

Thanks for the suggestions. We have fixed the problem and now the users can visualize Hi-Cdata at whole chromosome level.

**- It is also less dynamic than other tools such as Juicebox and HiGlass, but is maybe more dedicated to the exploration of single locus of interest. This should also be discussed.**

Thanks for the suggestion. We also added the following sentence in the discussion section.

“We also want to note that our browser is designed as query-based to maximize its usability, and as a result, it excels at exploring locus of interest and gene-element relationship, but a little less dynamic than other tools when navigating Hi-C matrix for larger genomic regions.”

**- All data are not always available. It would be great to have all normalization/all resolutions maps for all dataset. I did not tested all the dataset, but I only found normalized (iced) data for Human A549**

This is a great suggestion. We have now added the ICE (iterative correction and eigenvector decomposition) normalized matrices for all the Hi-C datasets. Normalization was done using the “iced” Python package based on Imakaev M et al. 2012. We will add normalized matrix along with raw matrix in the future when we add new datasets in our browser.

**- In the same way, I also looked at Mouse Neuron cells, but did not find TADs information ? If having all information for all samples is not possible, a table which summarizes which data is available for which dataset could be useful.**

This is another great suggestion. Now we have added 59 TAD tracks for all datasets re-aligned to GRCh38 and mm10 references, including the TADs information for Mouse Neuron cells (in

mm10). As stated above, we will predict and visualize the TAD information in the future as well when newer Hi-C data is added.
